# Antifibrotic therapies to control cardiac fibrosis

**DOI:** 10.1186/s40824-016-0060-8

**Published:** 2016-05-25

**Authors:** Zhaobo Fan, Jianjun Guan

**Affiliations:** Department of Materials Science and Engineering, The Ohio State University, 2041 College Road, Columbus, OH 43210 USA

**Keywords:** Myocardial infarction, Cardiac fibrosis, Myofibroblasts, Cardiac fibroblasts, Antifibrotic therapy

## Abstract

Cardiac fibrosis occurs naturally after myocardial infarction. While the initially formed fibrotic tissue prevents the infarcted heart tissue from rupture, the progression of cardiac fibrosis continuously expands the size of fibrotic tissue and causes cardiac function decrease. Cardiac fibrosis eventually evolves the infarcted hearts into heart failure. Inhibiting cardiac fibrosis from progressing is critical to prevent heart failure. However, there is no efficient therapeutic approach currently available. Myofibroblasts are primarily responsible for cardiac fibrosis. They are formed by cardiac fibroblast differentiation, fibrocyte differentiation, epithelial to mesenchymal transdifferentiation, and endothelial to mesenchymal transition, driven by cytokines such as transforming growth factor beta (TGF-β), angiotensin II and platelet-derived growth factor (PDGF). The approaches that inhibit myofibroblast formation have been demonstrated to prevent cardiac fibrosis, including systemic delivery of antifibrotic drugs, localized delivery of biomaterials, localized delivery of biomaterials and antifibrotic drugs, and localized delivery of cells using biomaterials. This review addresses current progresses in cardiac fibrosis therapies.

## Background

Myocardial infarction (MI) is the leading cause of death in the western countries. Cardiac fibrosis naturally occurs following MI. It is characterized by the excessive deposition of extracellular matrix (ECM) typically collagen in the infarcted area. Cardiac fibrosis increases stiffness and decreases compliance of the infarcted heart tissue. This negatively affects both contraction and relaxation behavior of the heart, resulting in a decrease in cardiac function. While the fibrotic tissue that forms initially may protect the heart from rupture, it gradually expands to the non-infarcted area when the MI evolves from early to late stages. The continuous increase of cardiac fibrosis leads to a progressive decrease in heart tissue contractility [1–5], and finally causes heart failure [6–8]. Cardiac fibrosis occurs not only after MI, but also from congenital defects, dilated cardiomyopathy and hypertension [9].

A therapy that can inhibit cardiac fibrosis from progressing in the infarcted hearts will preserve cardiac function and prevent heart failure. However, there is currently no efficient therapies available. Myofibroblasts are widely accepted to be responsible for cardiac fibrosis. They secrete excessive ECM directly leads to the formation of scar tissue. They also express highly contractile protein α-smooth muscle actin (αSMA) that remodels the surrounding ECM [10]. Understanding the origin of myofibroblasts may help to develop approaches to control tissue fibrosis.

## Sources of myofibroblasts in infarcted hearts

### Cardiac fibroblasts differentiation into myofibroblasts

**Fig. 1:**
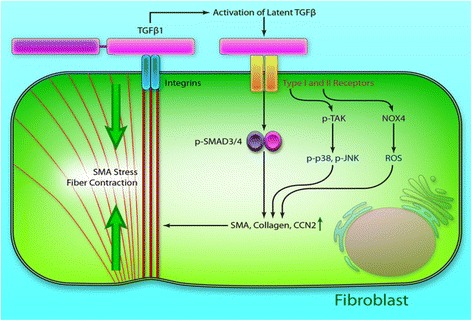
TGF-β signaling in fibroblasts. Latent TGF-β binds to its type I and II receptors, and activates the canonical Smad3/4 pathway and the noncanonical TGF-β–activated kinase-1 (TAK1)/p38/c-Jun N-terminal kinase (JNK) and NADPH oxidase 4 (NOX4)/reactive oxygen species (ROS) pathway resulting in induction of fibrogenic genes, such as α-smooth muscle actin (α-SMA) and collagen. (Reprinted from Leask A. [10])

Cardiac fibroblasts have greater quantities than cardiomyocytes in the heart tissue [11]. They are quiescent in healthy heart tissue, and are responsible for ECM secretion to keep the integrity of the interstitial matrix. They can also transduce survival signals and therefore control the conduction of electrical and mechanical stimuli to help maintaining the systolic and diastolic function in the heart tissue. Cardiac fibroblasts quickly respond to the changes to the surrounding microenvironment. After MI, the death of cardiomyocytes activates immune response that induces cytokine and chemokine expression. This initiates the infiltration of neutrophils and mononuclear cells to the infarcted area. The neutrophils are then phagocytosed by macrophages after apoptosis. The macrophages are able to secrete profibrotic cytokines like transforming growth factor beta (TGF-β), Angiotensin II, and platelet-derived growth factor (PDGF) [12]. TGF-β binds to TGF-β receptors type I and II, and activates the TGF-β/Smad pathway to differentiate the cardiac fibroblasts into myofibroblasts (Fig. 1) [10, 12]. TGF-β has been demonstrated as a major mediator of myofibroblast formation after MI. The formed myofibroblasts then produce excessive ECM to initiate the cardiac fibrosis. The myofibroblasts also secrete cytokines such as TGF-β, Angiotensin II, PDGF, tumor necrosis factor alpha (TNFα), and interleukin 1 beta (IL-1β) to further enhance the differentiation of cardiac fibroblasts into myofibroblasts. Angiotensin-II and PDGF indirectly promote myofibroblast differentiation by increasing TGF-β secretion [10].

Following myofibroblast differentiation, its number increases over a period of a few months in the infarcted area. More ECM is thus generated and deposited, leading to the increase of scar size. In the scar, the content of collagen type III-rich fibers increases in a few weeks. The fibers are then gradually replaced by stiffer type I collagen. The scar tissue matures when the collagen fibers are crosslinked. Unlike other scar tissues, myofibroblasts exist in the cardiac scar for many years, and continue generating ECM [13].

### Fibrocytes differentiation into myofibroblasts

Fibrocytes are a type of fibroblast-like peripheral cells [14]. These cells express fibroblast specific proteins, cluster of differentiation 31 and 45 (CD34 and CD45). In response to chemokines such as chemokine (C-C motif) ligand 21(CCL21) and chemokine (C-X-C motif) ligand 12 (CXCL12), fibrocytes migrate towards the injured area [15]. Under the stimulation of TGF-β or endothelin-1, fibrocytes differentiate into myofibroblast-type cells with expression of α-SMA, production of fibronectin and collagen, and loss of expression of CD34 and CD45 [16]. Besides TGF-β and endothelin-1, cytokines including IL-13, IL-14 and PDGF also promote the fibrocytes to differentiate into myofibroblasts [15, 17].

### Epithelial to mesenchymal transdifferentiation

Epithelial to mesenchymal transdifferentiation (EMT) is another origin of myofibroblasts, which is a process of transdifferentiation from epithelial cells into myofibroblast-like cells. In the EMT process, the expression of mesenchymal marker is up-regulated while the expression of epithelial marker is down-regulated [18, 19]. TGF-β1 plays a key role in this process. While the roles of other cytokines in EMT are still in debate, strong evidences suggest that TNFα and IL-1β are capable of accentuating the effect of TGF-β1 in driving EMT [20, 21].

### Endothelial to mesenchymal transition

Endothelial to mesenchymal transition (EnMT) is currently thought to be a potential origin of myofibroblasts. EnMT was first proposed to be a phenomenon related to embryonic development until evidence indicated that up to 35 % of fibroblasts in fibrotic heart muscle are converted from endothelial cells [22]. Similar to EMT, EnMT can be driven by TGF-β (Types 1 and 2) and be augmented by TNFα and IL-1β [22–24].

## Therapies for cardiac fibrosis

After MI, myofibroblasts differentiated from cardiac fibroblasts are mainly responsible for cardiac fibrosis. Therefore, control of cardiac fibroblast differentiation into myofibroblasts is critical to attenuate cardiac fibrosis. As discussed above, growth factors and cytokines like TGF-β, Angiotensin-II, and PDGF directly and indirectly involve in myofibroblast differentiation. Current therapies are thus focused on reducing the secretion of these growth factors and cytokines, and decreasing the amount of active growth factors and cytokines. These therapies include systemic delivery of antifibrotic agents, localized delivery of biomaterials, localized delivery of biomaterials and antifibrotic agents, and localized delivery of biomaterials and stem cells.

### Systemic delivery of antifibrotic agents

Antifibrotic agents that decrease the activity or level of growth factors and cytokines such as TGF-β, Angiotensin-II, PDGF, TNFα and IL-1β can decrease myofibroblast activation, thus decreasing cardiac fibrosis. Since TGF-β is considered as a major mediator for cardiac fibroblasts to differentiate into myofibroblasts, inhibiting TGF-β from attacking cardiac fibroblasts and decreasing the amount of active TGF-β in the infarcted hearts may decrease the number of myofibroblasts. TGF-β receptor type I (ALK5) inhibitors like GW 788388 have been developed to decrease TGF-β activity [25]. Anti-TGF-β antibodies decrease the amount of active TGF-β [10]. In addition, angiotensin-converting enzyme (ACE) inhibitors are associated with reducing TGF-β level [26, 27].

In cardiac fibroblasts, Angiotensin-II induces expression of TGF-β1 through angiotensin type-I receptor [28]. It also induces expression of collagen through TGF-β/Smad pathway and extracellular signal-regulated kinase by IL-6 dependent mechanism [29, 30]. Angiotensin receptor inhibitors like losartan have been shown to reduce cardiac fibrosis in animal and human trials [31, 32]. Inflammation plays a role in the formation and progression of cardiac fibrosis. The cytokines released from macrophages and T cells, such as IL-1β and TNF-α, can promote the proliferation of cardiac fibroblasts and upregulate the expression of tissue inhibitor of matrix metalloproteinase (TIMP)-1, leading to cardiac fibrosis [33]. Use of drugs to suppress inflammation in the heart has shown benefits in reducing cardiac fibrosis. For example the administration of the selective p38 MAPK inhibitor blocked the secretion of TNFα and decreased cardiac fibrosis [34]. The drugs inhibiting cardiac fibroblast growth can also be used to inhibit cardiac fibrosis, such as β-blockers, relaxin, and statins [35–37].

The above drugs for control of cardiac fibrosis are generally administrated by a systemic delivery approach through either oral intake or injection. The major advantage of this approach is that it is convenient. Yet the drug dosage allocated to the infarcted heart may be low, which decreases the therapeutic efficacy. Increase of the initial drug dosage may cause toxic effect. In addition, the drugs allocated to other tissues may have side effects on these tissues and cells inside. Localized delivery of antifibrotic drugs has the potential to address those disadvantages.

### Localized delivery of biomaterials

Biomaterials can be used to control cardiac fibrosis. These biomaterials include naturally-derived matrices such as collagen [38] and alginate [39], and synthetic biomaterials such as poly(N-isopropyl acrylamide)-based hydrogels [40]. Besides antifibrotic properties, these biomaterials provide mechanical support to the infarcted tissue and decrease elevated wall stress, resulting in improved cardiac function [41].

Decellularized cardiac ECM is a naturally-derived matrix that has shown promise for treating infarcted hearts after MI [42]. It provides cells with tissue specific biochemical cues important for cell migration and differentiation, and tissue regeneration [42]. The major composition of decellularized cardiac ECM includes collagen, elastin, fibronectin, and GAGs. While fibronectin has been shown to promote cardiac fibroblasts to differentiate into myofibroblasts to facilitate cardiac fibrosis [43], the growth factors retained in the matrix such as hepatocyte growth factor (HGF) may inhibit this differentiation [44]. It is also possible that decellularized ECM increases MMP-1 secretion in the infarcted hearts, thus decreasing collagen deposition [45]. Injection of the hydrogel based on decellularized porcine cardiac ECM into rat MI model significantly decreased fibrosis in infarcted area [46].

In the infarcted hearts, elevated wall stress resulting from left ventricle dilation represents a powerful stimulus for intracellular signaling transduced by mechanoreceptors [47, 48]. The increased wall stress leads to the activation of local tissue renin-angiotensin system, causing up-regulation of angiotensin II. The upregulated angiotensin II increases tissue inflammation, and TGF-β, IL-1β and TNF-α secretion [49–51]. These events lead to the enhanced formation of myofibroblasts. Therefore, use of biomaterials that can effectively decrease wall stress will decrease tissue inflammation, resulting in decreased cardiac fibrosis. These biomaterials are soft with modulus typically similar to or lower than that of the heart tissue. On the other hand, biomaterials implantation is always associated with foreign-body response and inflammation, which may compromise the effect from reduced wall stress. The choose of biomaterials that cause less inflammation is thus critical.

Hydrogel based on alginate and chitosan has been shown to decrease cardiac fibrosis [39]. The hydrogel was highly soft with mean storage modulus of 20 ± 15 Pa. Injection of this hydrogel into infarcted rat hearts significantly increased wall thickness, leading to the decrease of wall stress. As a result, the number of CD68+ macrophages was significantly decreased compared to phosphate-buffered saline (PBS) injection [39]. The reduced tissue inflammation largely decreased tissue fibrosis after 8 weeks. In addition, injection of alginate and chitosan enhanced tissue vascularization. The reduced cardiac fibrosis and enhanced vascularization significantly increased cardiac function. Similar results were found when injecting soft collagen and poly(N-isopropylacryamide-co-2-hydroxyl methacrylate-co-methacrylate- polylactide) hydrogels into infarcted hearts [38, 47].

**Fig. 2:**
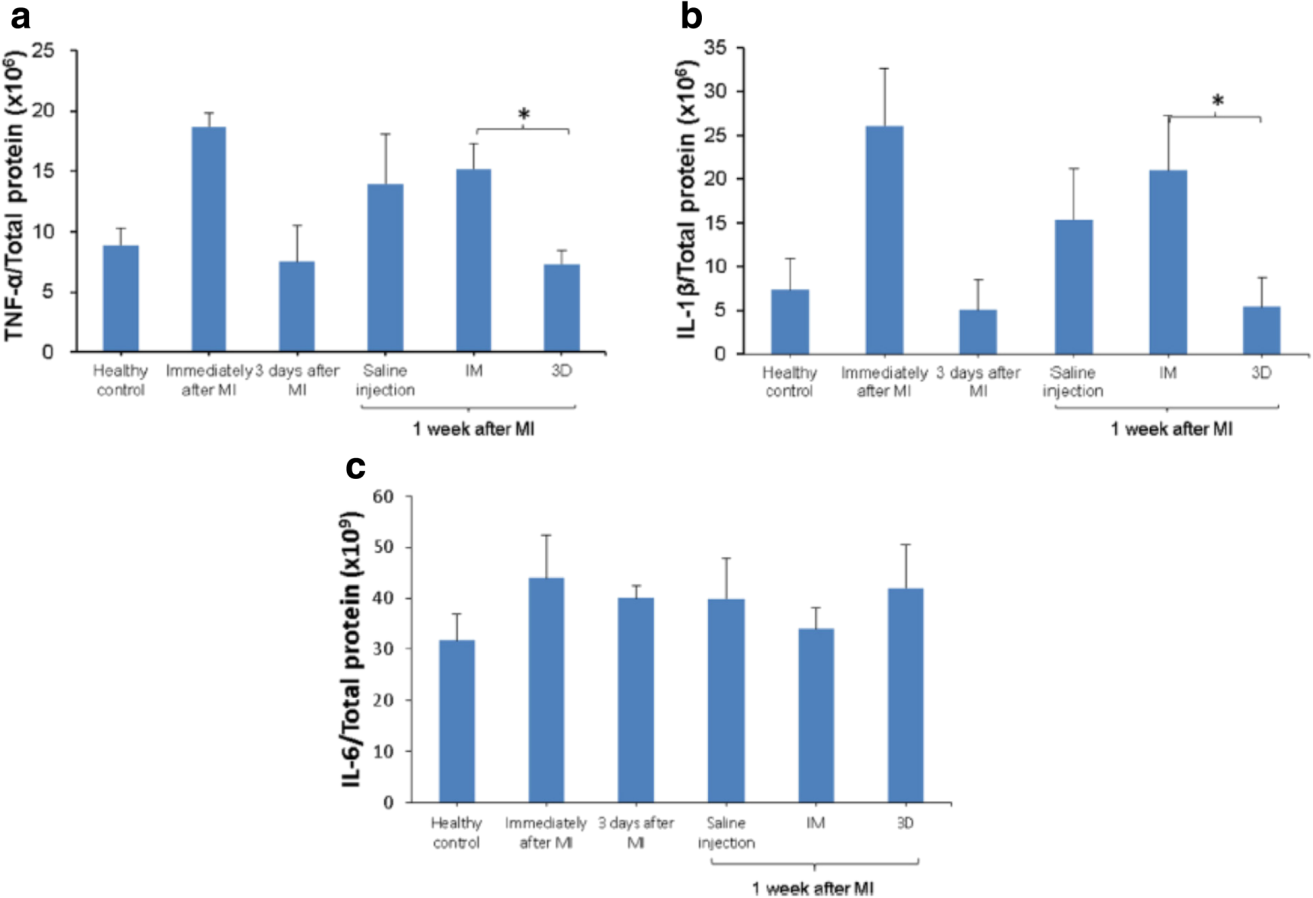
Effect of time and hydrogel injection on the expression of TNF-α (**a**), IL-1β (**b**) and IL-6 (**c**) in infarcted left ventricle. * indicates significant differences between groups. Rats were divided into 3 injection treatment groups (immediately after MI (IM), 3 d after MI (3D) and 2 w after MI (2 W)) and 2 control groups (healthy and MI without treatments). (Reprinted from Yoshizumi et al. [47])

**Fig. 3:**
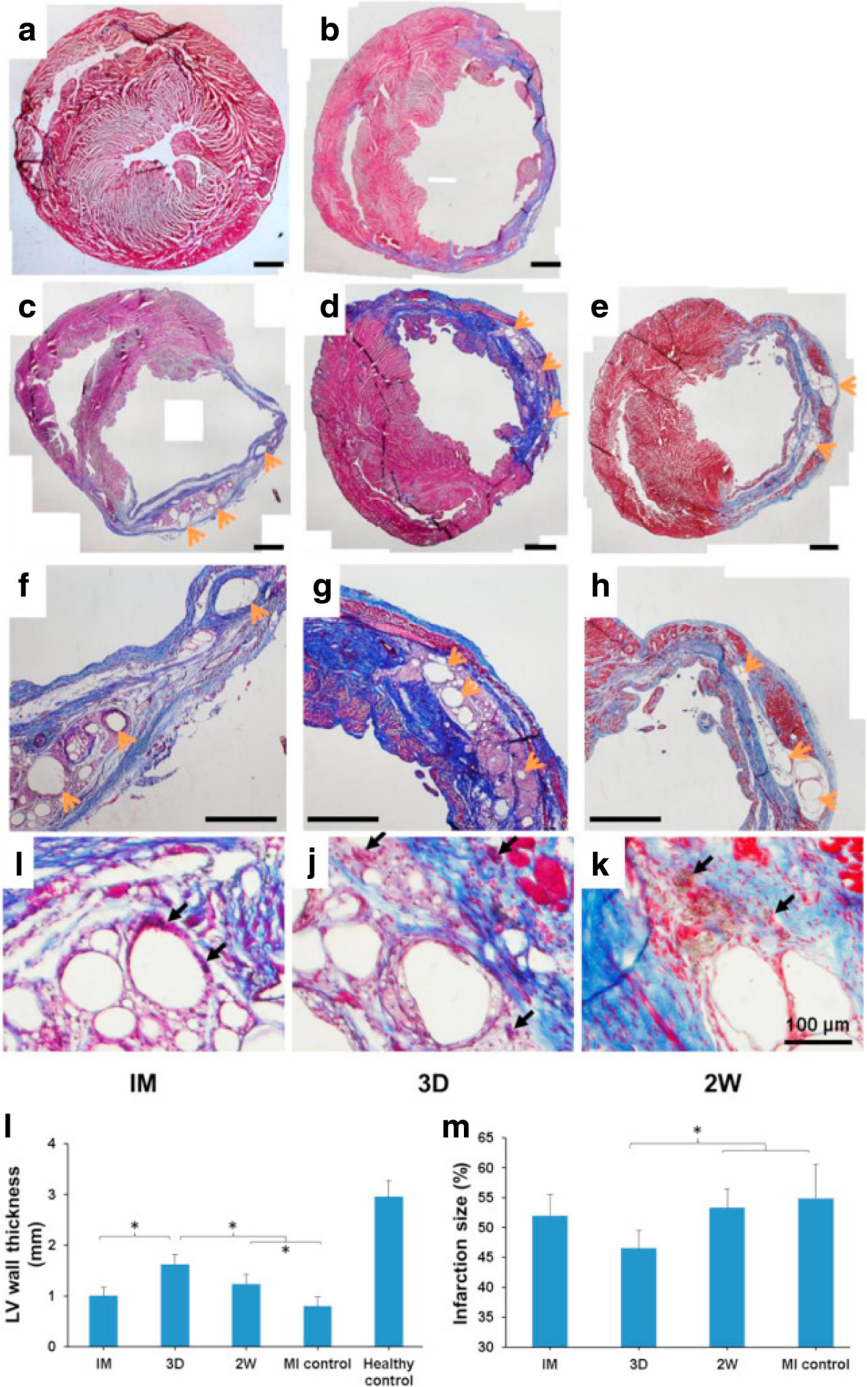
Ventricular wall histology for rat hearts 10 w after MI. Rats were divided into 3 injection treatment groups (immediately after MI (IM), 3 d after MI (3D) and 2 w after MI (2 W)) and 2 control groups (healthy and MI without treatments). Representative Masson’s trichrome stained cross-sections: **a** Healthy control, **b** MI control, **c**, **f**, **i** IM group, **d**, **g**, **j** 3D group, **e**, **h**, **k** 2 W group. A-H scale bars = 1 mm. Orange arrows point to the hydrogel residues, black arrows point to foreign body giant cells. Wall thickness (**l**) and infarction size (**m**) were measured from the complete set of these images. * indicates significant differences between groups. (Reprinted from Yoshizumi et al. [47])

When using hydrogels to decrease cardiac fibrosis, time of the injection affects therapeutic efficacy [38, 47]. This is because wall stress of the infarcted tissue varies when the MI evolves from early to late stages. In general, the wall stress gradually increases from the necrotic phase to the fibrotic phase [52]. Therefore, the same hydrogel may have different efficacy in reducing wall stress and inflammation. In addition, the inflammation at different stage of MI is different with the most severe inflammation observed at the beginning of the MI [47]. Yoshizumi et al. found that the degree of inflammation was significantly higher in hearts injected with the hydrogel immediately after MI than in those injected with hydrogel 3 days after MI [47]. As a result, the hearts injected with the hydrogel 3 days after MI showed the lowest cardiac fibrosis. When injecting the hydrogels 2 weeks after MI where the cardiac fibrosis was already formed, the hydrogels did not decrease cardiac fibrosis to the extent when they were injected 3 days after MI (Figs. 2 and 3). The above results suggest that timing of hydrogel injection should be considered in order to achieve optimal therapeutic effect.

### Localized delivery of biomaterials and antifibrotic agents

Localized delivery of antifibrotic agents has the potential to address the low efficacy issue associated with the systemic delivery as the dosage in the infarcted area is higher. Yet the inherent disadvantage is that repeated delivery by open surgery is impractical. Delivery of drugs using minimally invasive surgery can avoid this issue. However, repeated delivery increases cost for the therapy and thereby is not ideal. These disadvantages may be overcome by using drug delivery systems that continuously release drugs. In these systems, the drugs are encapsulated in injectable biomaterials such as hydrogels and microspheres. The drugs then gradually release from the biomaterials by diffusion and biomaterial degradation. In addition, the biomaterials can increase drug retention in the heart tissue.

Sustained delivery of drugs that decrease inflammation in the infarcted hearts after MI may decrease the inflammation cytokines-associated myofibroblast formation, thereby reducing cardiac fibrosis. Ibuprofen is a cyclooxygenase inhibitor with anti-inflammatory property. It directly inhibits leukocyte activation and accumulations, and decreases the production of leukocyte attractant and activator leukotriene B4 [53]. Vu et al. injected ibuprofen-containing hyaluronic acid hydrogel into infarcted pig hearts, and found that cardiac fibrosis was significantly decreased compared to the hydrogel only group [53]. Erythropoietin (EPO) shows a cardioprotective effect after acute MI [54]. It augments cell survival in the infarcted hearts by activation of prosurvival signals Stat3, Akt, and ERK. Stat3 has been found to be closely related to tissue inflammation. A deletion of Stat3 is susceptible to dramatic increase of inflammation-induced cardiac fibrosis [54]. The activation of stat3 is thus expected to decrease cardiac fibrosis. Kobayashi et al. encapsulated EPO in the gelatin hydrogel and implanted into infarcted hearts after acute MI [54]. The EPO was able to gradually release from the hydrogel for 14 days. The released EPO significantly decreased cardiac fibrosis and infarct size 14 days and 2 months after MI, leading to the increase in cardiac function.

Delivery of antifibrotic growth factors represents an effective approach to attenuate cardiac fibrosis. One of the strong candidates is HGF. It is a potent agonist for the tyrosine kinase surface receptor c-MET. HGF exerts its antifibrotic property in two ways: inhibition of collagen synthesis by suppression of TGFβ expression, and degradation of collagen by activation of matrix metalloproteinase-1 (MMP-1) [55]. In addition, HGF may attenuate inflammation in the tissue, thereby decreasing inflammation-associated myofibroblast formation [56]. Besides antifibrotic property, HGF has pro-angiogenic, anti-apoptotic and cardioprotective activity [44, 55, 57–60]. Therefore, HGF is an attractive growth factor for cardiac therapy. Taniyama et al. transfected HGF gene in the cardiomyopathic hamsters and found that cardiac fibrosis was significantly reduced and angiogenesis was enhanced [55]. The disadvantage of gene transfection approach lies in safety concern and inflammation associated with viral vectors. Direct delivery of HGF may be an approach but has low efficacy due to its short half-life in solution form. Sustained delivery of HGF using biomaterials addresses this issue. Nakano et al. developed a HGF delivery system using porous crosslinked gelatin patch [61]. HGF was able to release from the scaffold over 2 weeks. After 2 and 4 weeks of implantation on the epicardium surface of the infarcted rat hearts, the fibrotic area was significantly decreased from 17.5 % (control, without implantation) to 8.8 %. The decrease of cardiac fibrosis increased cardiac function as fractional shortening and end-systolic elastance were significantly greater in the HGF treatment group.

Clinical application of HGF especially recombinant human HGF for cardiac therapy is obstructed by the high cost, challenging to manufacture and short half-life [62]. To increase the translational potential of HGF, Sonnenberg et al. engineered a HGF biomimetic - HGF fragment. The HGF fragment can be produced at a high yield and show similar potency as HGF [63, 64]. In addition, it is more stable than HGF. To deliver HGF fragment into infarcted heart, it was encapsulated into a hydrogel based on decellularized porcine epicardium. The HGF fragment exhibited slow release kinetics, resulting from the binding of HGF fragment with GAGs in the hydrogel [46]. After delivery into infarcted hearts, the released HGF fragment significantly downregulated TGFβ expression and upregulated MMP-1 expression in cardiac cells. Four weeks after injection, collagen content in the infarcted area was significantly decreased compared to control, indicating that the cardiac fibrosis was attenuated.

**Fig. 4:**
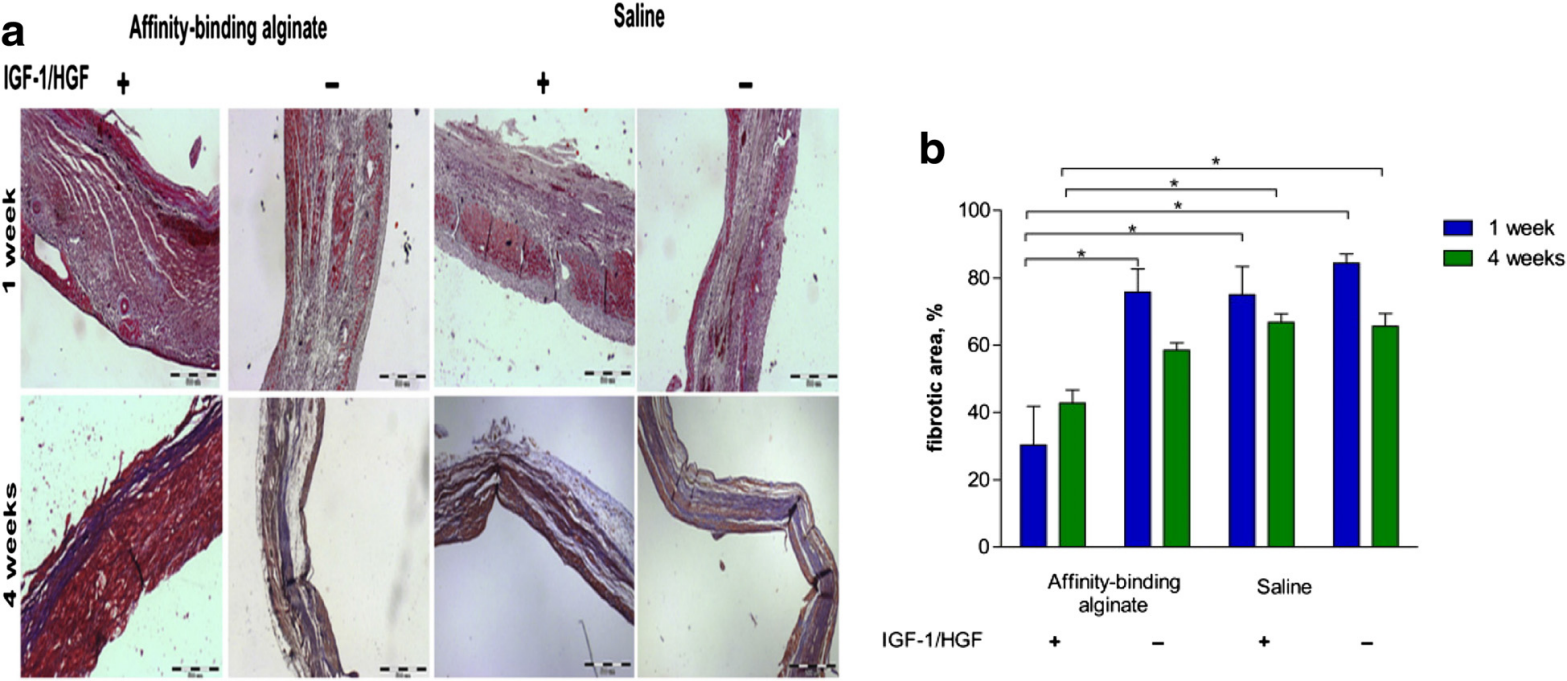
The sequential IGF-1/HGF delivery using alginate hydrogel reduces fibrosis. **a** Representative photomicrographs of Masson’s trichrome staining (collagen-rich areas in blue and healthy myocardium in red), scar area. Bar = 500 μm. **b** Fibrotic content of the scar. * *p* < 0.05. (Reprinted from Ruvinov et al. [66])

Co-delivery of HGF and other growth factors can also inhibit cardiac fibrosis. For example, HGF and insulin-like growth factor-1 (IGF-1) were co-delivered into infarcted hearts using alginate hydrogel as a carrier [65, 66]. IGF-1 has cytoprotective effect. It increases cell survival in the infarcted hearts. The decreased tissue apoptosis reduces fibrotic tissue formation. The advantage of dual release is that IGF-1 and HGF can be sequentially released from the hydrogel. HGF released slower than IGF-1 due to a much higher molecular weight. The first released IGF-1 promoted cell survival while the latter released HGF decreased fibrotic tissue formation and stimulated angiogenesis. Both growth factors were able to release from the alginate for 7 days. The amount of released IGF-1 was much higher than that of HGF [66]. The released growth factors remained bioactive. After being delivered into infarcted rat hearts for 4 weeks, the dual release group significantly decreased fibrotic area compared to the alginate only group (Fig. 4). The released growth factors also augmented cardiac cell survival.

Delivery of HGF together with vascular endothelial growth factor (VEGF) can also decrease cardiac fibrosis [67]. Salimath et al. encapsulated HGF and VEGF into collagenase degradable PEG hydrogel [67]. When incubated in PBS, both growth factors exhibited nearly linear release from the hydrogel. In collagenase solution, complete release was achieved in 4 days. After delivering the release system into infarcted hearts for 3 weeks, the non-treatment animals had fibrotic area of 41.5 % while those treated with VEGF and HGF had only 13.9 %. The combined VEGF and HGF treatment showed significantly higher efficacy than individual growth factor treatment. These results suggested that VEGF played a role in reducing cardiac fibrosis. This may be the result of VEGF promoting angiogenesis in the infarcted area [68, 69]. The vascularized tissue has reduced fibrosis. It is also possible that VEGF and HGF synchronously recruited progenitor cells for myocardial regeneration [67].

Basic fibroblast growth factor (bFGF) is another growth factor that has been demonstrated to inhibit cardiac fibrosis. The mechanism is that bFGF attenuates cardiac fibroblasts to differentiate into myofibroblasts in the presence of TGFβ [70]. While it is not clear whether this effect is resulted only from blocking TGFβ/Smad signaling pathway, studies based on valvular interstitial cells suggested that TGFβ/Erk1/2 pathway may be also involved [71–75]. One of the approaches to deliver bFGF into infarcted hearts is to encapsulate it into crosslinked albumin-alginate microcapsules followed by injection [76]. The released bFGF substantially decreased collagen content in the infarcted area. Co-delivery of bFGF with HGF further reduced collagen content. This study demonstrated that delivery of two antifibrotic growth factors may more efficiently attenuate cardiac fibrosis.

Studies have shown that Notch1 signaling plays a critical role in the cardiac fibroblast-myofibroblast transformation, thereby affecting cardiac fibrosis [77]. Notch1 activation is expected to inhibit the transformation and prevent cardiac fibrosis. This can be achieved by using Notch ligand Jagged-1 (peptide CDDYYYGFGCNKFCRPR) [78]. To deliver the ligand into heart, it was encapsulated into a self-assembling peptide-based hydrogel (peptide RARADADARARADADA). Three weeks after delivery, picrosirius red staining results demonstrated that the fibrotic area was significantly decreased compared to self-assembling peptide control. Besides preventing cardiac fibrosis, the Notch ligand also improved angiogenesis, induced cardiac cell proliferation and stem cell recruitment. Compared with growth factors like HGF, VEGF, IGF-1 and bFGF, the peptide based Notch ligand is less expensive yet possesses the function of these growth factors in terms of prosurvival, proangiogenic, and antifibrotic properties. Therefore, the peptide based cardiac therapy may have greater translational potential.

### Localized delivery of biomaterials and cells

Cell therapy represents a promising approach to treat cardiac fibrosis. It may also lead to heart tissue vascularization and regeneration. In fact, tissue vascularization and regeneration can decrease fibrotic tissue area. Various cell types have been explored in clinical and preclinical models for cardiac therapy. Some stem cell types do not differentiate into cardiomyocytes to directly regenerate cardiac tissue, but can indirectly promote the regeneration. These cell types typically provide paracrine effects to inhibit cardiac fibrosis, augment the survival of resident cardiac cells, recruit endogenous stem cells, and vascularize the damaged heart tissue [41, 79–84]. Some cell types also directly participate in tissue vascularization. Examples include bone marrow mesenchymal stem cells (BMMSCs) [85–99] and adipose-derived stem cells (ADSCs) [100–103]. Cardiomyocytes and stem cells capable of differentiating into cardiomyocytes can be used to regenerate cardiac tissue. These stem cells include cardiac stem/progenitor cells [104–108], pluripotent stem cell [embryonic stem cells (ESCs) and induced pluripotent stem cells (iPSCs)]-derived cardiovascular progenitor cells [109–111], and cardiosphere-derived cells [112–122]. To deliver cells into infarcted hearts, direct injection experiences low efficacy because of inferior cell retention in the tissue. To increase cell retention, cells can be encapsulated into injectable materials such as hydrogels or microspheres. The cells can also be loaded into porous scaffolds or hydrogels and then patch on the tissue surface.

**Fig. 5:**
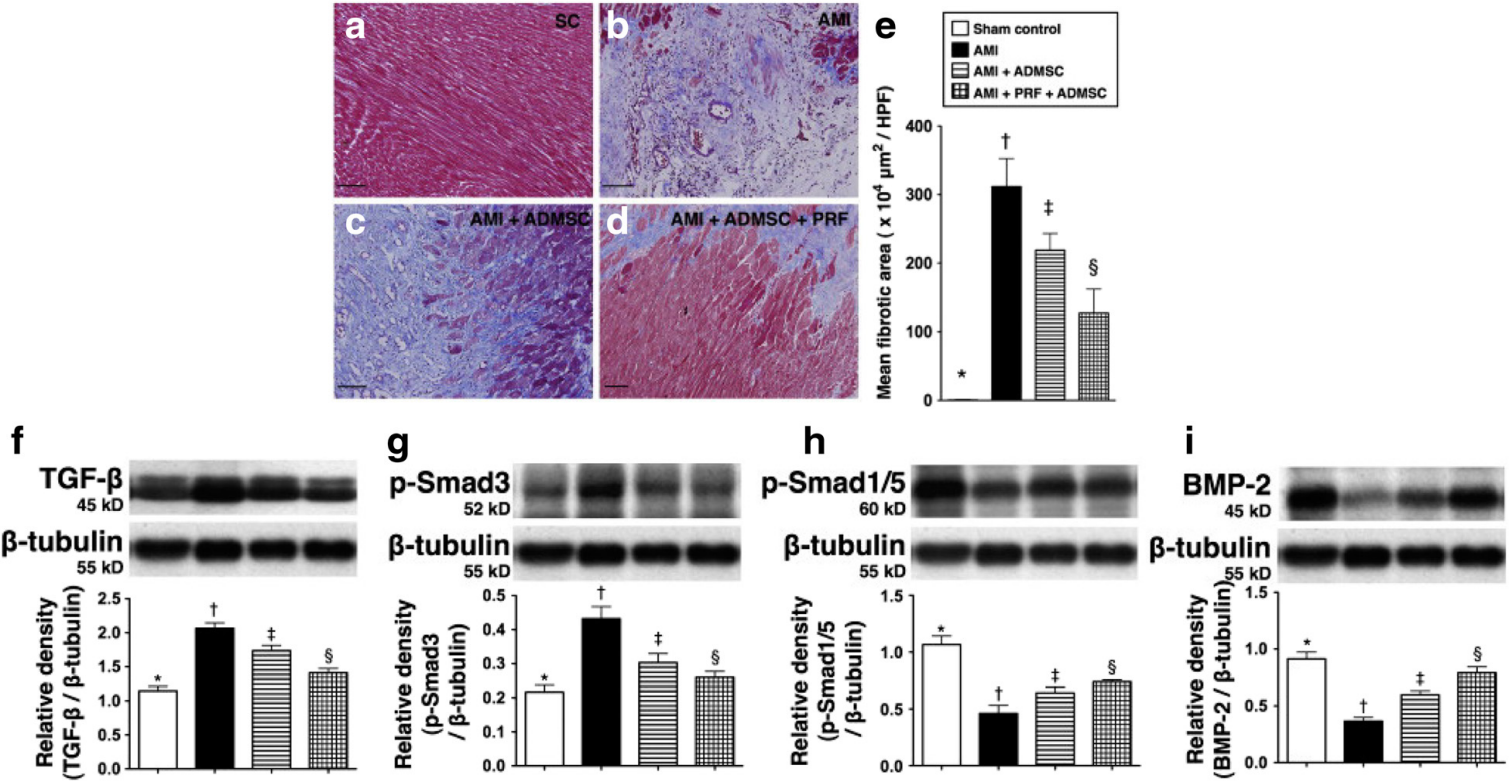
Left ventricular (LV) myocardial histopathological changes on post-infarct day 42. **a** to **d** Microscopic identification of myocardial fibrosis over LV after Masson’s Trichrome staining. **e** Mean fibrotic area. **f**–**i** Protein expressions of fibrotic (TGF-β and Smad3) and antifibrotic (Smad1/5 and BBMP-2) biomarkers in infarct area of LV myocardium. * vs. other groups with different symbols (*, †, ‡, §), *p* < 0.05 for all groups. AMI: acute MI. ADMSC: adipose derived MSC. PRF: platelet-rich fibrin. (Reprinted from Sun et al. [123])

For those stem cells that indirectly promote heart tissue regeneration, the cell paracrine effects may contribute to decreased fibrosis by decreasing inflammation and profibrotic factor expression. Sun et al. encapsulated ADSCs into platelet-rich fibrin and then patched on the surface of infarcted hearts. Use of fibrin significantly increased cell survival and paracrine effects, which led to the significant decrease of the expression of fibrotic mediators TGF-β and Smad3 (Fig. 5). In addition, the expression of antifibrotic markers Smad1/5 and BMP-2 was significantly increased (Fig. 5) [123]. The enhanced cell survival and paracrine effects also decreased the expression of inflammation markers CD3, CD40, CD68 and CD19. As a result, the fibrotic area was significantly decreased as determined by Masson’s Trichrome staining (Fig. 5). When transplanting ADSCs in collagen patches, the level of procollagen C-proteinase that is responsible for procollagen processing into mature collagen was significantly reduced. In addition, the level of lysyl oxidase, the enzyme involved in collagen crosslinking in the peri-infarct region, was decreased [124]. These events led to the decrease of cardiac fibrosis.

BMMSCs also contribute to decreased cardiac fibrosis. BMMSCs have been found to attenuate cardiac fibroblast proliferation and collagen synthesis through paracrine effects [125]. While it remains unclear how paracrine effects decrease cardiac fibroblast proliferation, the paracrine effects do downregulate the expression of Col1a1 and Col3a1 [125]. BMMSCs also promote cardiac fibroblasts to secrete matrix metalloproteinases that degrade collagen [98]. Transplantation of BMMSCs into infarcted hearts can attenuate cardiac fibrosis at different stages of MI [126–128]. At the acute stage, the cardiac fibrosis is not formed while at later stages it is formed and progresses with time. Therefore, control of cardiac fibrosis at acute MI stage may more efficiently attenuate cardiac fibrosis. Ceccaldi et al. seeded BMMSCs into microporous alginate-chitosan scaffolds and implanted the constructs on infarcted area using acute MI model [128]. After 33 days, the fibrosis percentage was decreased more in the BMMSCs seeded scaffolds than in the pure scaffolds. Similar results were found when injecting BMMSCs-encapsulated hydrogel into infarcted mice hearts after acute MI. Xia et al. encapsulated BMMSCs into a thermosensitive hydrogel based on N-isopropylacrylamide/acrylic acid and 2-hydroxyletheyl methacrylate-polycaprolactone [126]. After 28 days of implantation, the collagen content in the scar tissue was significantly decreased compared to transplantation of BMMSC only. The decease of fibrosis may be resulted from increased cell survival in the hydrogel, which provided greater paracrine effects to inhibit cardiac fibrosis. BMMSC transplantation can also decrease cardiac fibrosis when the MI is in the subacute stage. Fiumana et al. seeded BMMSCs into porous hyaluronan-based scaffolds and placed on the top of the infarcted hearts two weeks following MI [128]. After two weeks of implantation, cardiac fibrosis was largely attenuated.

Implantation of cardiomyocytes that promote cardiac tissue regeneration also has the potential to decrease cardiac fibrosis. Joanne et al. seeded induced pluripotent stem cells-derived cardiomyocytes (iPSC-CMs) into collagen scaffolds with modulus similar to that of the native heart tissue (10–15 kPa) [129]. After 3 days of in vitro culture, the constructs were patched on the dilated mouse hearts. Following 2 weeks of implantation, the transplanted iPSC-CMs integrated with the native myocardium and contributed to the cardiac fibrosis decrease. The hearts implanted with constructs showed significantly higher expression of osteopontin that regulates matrix metalloproteinases than those implanted with pure collagen scaffolds.

## Conclusions

Control of cardiac fibrosis is essential to prevent the infarcted hearts from progressing into heart failure. The cardiac fibrosis should ideally be controlled immediately after MI so that the processes that initiate the cardiac fibrosis can be inactivated. Yet the heart tissue may rupture without the protection of the initial fibrotic layer. Cardiac fibrosis naturally expands upon the initial fibrotic tissue is formed. Inhibiting cardiac fibrosis from progressing may prevent progressive deterioration of cardiac function. Different approaches have been explored to treat cardiac fibrosis, such as systemic delivery of antifibrotic drugs, localized transplantation of biomaterials, localized delivery of antifibrotic drugs using biomaterials, and localized delivery of cells and biomaterials. Compared to localized delivery approaches, the systemic delivery approach is more convenient. However, the drug dosage allocated to the heart is limited, resulting in lower therapeutic efficacy.

Localized transplantation of biomaterials controls cardiac fibrosis by decreasing left ventricular wall stress to decrease the elevated wall stress-induced inflammation. When using decellularized ECM, the growth factor released from the matrix may also decrease cardiac fibrosis. Selection of biomaterials with suitable mechanical properties is critical to decrease wall stress. The ideal biomaterials should have elasticity and stiffness matching those of the heart tissue. During the biomaterial degradation, these mechanical properties may decrease. Yet the cells from surrounding tissue may penetrate into the biomaterials to vascularize the tissue, and promote regeneration. Besides mechanical properties, the biomaterials should have excellent biocompatibility without provoking significant inflammation.

Localized delivery of drugs and biomaterials exhibited higher efficacy in controlling cardiac fibrosis than delivery of biomaterials only. The drugs and biomaterials may be delivered into infarcted hearts shortly after MI and before the initial fibrotic tissue is formed since the biomaterials may provide adequate mechanical support to prevent tissue rupture. The encapsulated drugs gradually release from the biomaterials allowing for long-term attenuation of cardiac fibrosis. The efficacy of cardiac fibrosis inhibition is determined by drug release kinetics. When the released drug is sufficient to prevent new myofibroblast formation and ECM synthesis especially collagen, high efficacy can be achieved. Duration of drug release also determine the therapeutic efficacy. Longer time delivery better controls cardiac fibrosis. Thus tailoring properties of the biomaterials to enable the drugs to release for prolonged time period is essential.

Localized delivery of cells using biomaterials has been shown to be an effective approach to control cardiac fibrosis. The cells either provide paracrine effects or directly regenerate the infarcted heart tissue. Those cells that provide paracrine effects may release antifibrotic factors to directly control cardiac fibrosis. They may release anti-inflammatory factors to control inflammation thus indirectly controlling cardiac fibrosis. In addition, the released angiogenic factors promote tissue vascularization and regeneration. To long-term control cardiac fibrosis, high rate of cell survival in the infarcted hearts are necessary. However, the infarcted heart tissue is characterized by a low nutrient and oxygen environment. The delivered cells thus experience low survival rate. The inflammation condition in the infarcted hearts also causes cell death. Use of biomaterials as cell carriers may increase the cell survival by protecting the cells from attack by inflammatory cytokines, but do not necessarily improve cell survival under the low nutrient and oxygen conditions. Approaches that can be used to increase cell survival under these conditions include encapsulation of cells in biomaterials that release prosurvival growth factors such as bFGF, IGF-1 and HGF [130–135], and release oxygen [136].

In summary, different approaches have been used to inhibit cardiac fibrosis. Localized drug delivery represents a major approach. Yet the widespread clinical application of current approaches is obstructed by the low therapeutic efficacy. Development of new and translational delivery approaches to improve therapeutic efficacy is essential to push the anti-cardiac fibrosis therapy towards clinical application. In addition, development of new drugs that not only prevent myofibroblast formation but also revert existing myofibroblasts back into the cardiac fibroblasts may fundamentally prevent cardiac fibrosis.

## References

[1] Dobaczewski M, de Haan JJ, Frangogiannis NG (2012). The extracellular matrix modulates fibroblast phenotype and function in the infarcted myocardium. J Cardiovasc Transl Res.

[2] Gonzalez A, Ravassa S, Beaumont J, Lopez B, Diez J (2011). New targets to treat the structural remodeling of the myocardium. J Am Coll Cardiol.

[3] van den Borne SW, Diez J, Blankesteijn WM, Verjans J, Hofstra L, Narula J (2010). Myocardial remodeling after infarction: the role of myofibroblasts. Nat Rev Cardiol.

[4] Yarbrough WM, Mukherjee R, Stroud RE, Rivers WT, Oelsen JM, Dixon JA (2012). Progressive induction of left ventricular pressure overload in a large animal model elicits myocardial remodeling and a unique matrix signature. J Thorac Cardiovasc Surg.

[5] Kong P, Christia P, Frangogiannis NG (2014). The pathogenesis of cardiac fibrosis. Cell Mol Life Sci.

[6] Janicki JS, Brower GL, Gardner JD, Chancey AL, Stewart JA (2004). The dynamic interaction between matrix metalloproteinase activity and adverse myocardial remodeling. Heart Fail Rev.

[7] Spinale FG, Janicki JS, Zile MR (2013). Membrane-associated matrix proteolysis and heart failure. Circ Res.

[8] Tsuruda T, Costello-Boerrigter LC, Burnett JC (2004). Matrix metalloproteinases: pathways of induction by bioactive molecules. Heart Fail Rev.

[9] Creemers EE, Pinto YM (2011). Molecular mechanisms that control interstitial fibrosis in the pressure-overloaded heart. Cardiovasc Res.

[10] Leask A (2015). Getting to the heart of the matter: new insights into cardiac fibrosis. Circ Res.

[11] Nag AC (1980). Study of non-muscle cells of the adult mammalian heart: a fine structural analysis and distribution. Cytobios.

[12] Shinde AV, Frangogiannis NG (2014). Fibroblasts in myocardial infarction: a role in inflammation and repair. J Mol Cell Cardiol.

[13] Willems IE, Havenith MG, De Mey JG, Daemen MJ (1994). The alpha-smooth muscle actin-positive cells in healing human myocardial scars. Am J Pathol.

[14] Bucala R, Spiegel LA, Chesney J, Hogan M, Cerami A (1994). Circulating fibrocytes define a new leukocyte subpopulation that mediates tissue repair. Mol Med.

[15] Chesney J, Bucala R (1997). Peripheral blood fibrocytes: novel fibroblast-like cells that present antigen and mediate tissue repair. Biochem Soc Trans.

[16] Mori L, Bellini A, Stacey MA, Schmidt M, Mattoli S (2005). Fibrocytes contribute to the myofibroblast population in wounded skin and originate from the bone marrow. Exp Cell Res.

[17] Aiba S, Tagami H (1997). Inverse correlation between CD34 expression and proline-4-hydroxylase immunoreactivity on spindle cells noted in hypertrophic scars and keloids. J Cutan Pathol.

[18] Jain R, Shaul PW, Borok Z, Willis BC (2007). Endothelin-1 induces alveolar epithelial-mesenchymal transition through endothelin type A receptor-mediated production of TGF-beta1. Am J Respir Cell Mol Biol.

[19] Kalluri R, Neilson EG (2003). Epithelial-mesenchymal transition and its implications for fibrosis. J Clin Invest.

[20] Doerner AM, Zuraw BL (2009). TGF-beta1 induced epithelial to mesenchymal transition (EMT) in human bronchial epithelial cells is enhanced by IL-1beta but not abrogated by corticosteroids. Respir Res.

[21] Yamauchi Y, Kohyama T, Takizawa H, Kamitani S, Desaki M, Takami K (2010). Tumor necrosis factor-alpha enhances both epithelial-mesenchymal transition and cell contraction induced in A549 human alveolar epithelial cells by transforming growth factor-beta1. Exp Lung Res.

[22] Zeisberg EM, Tarnavski O, Zeisberg M, Dorfman AL, McMullen JR, Gustafsson E (2007). Endothelial-to-mesenchymal transition contributes to cardiac fibrosis. Nat Med.

[23] Maleszewska M, Moonen JR, Huijkman N, van de Sluis B, Krenning G, Harmsen MC (2013). IL-1beta and TGFbeta2 synergistically induce endothelial to mesenchymal transition in an NFkappaB-dependent manner. Immunobiology.

[24] Dai H, Huang H, Wang SL, Xu X, Jian Y, Cui WH (2012). Role of tumor necrosis factor alpha in endothelial-mesenchymal transition in vitro. Zhonghua Shao Shang Za Zhi.

[25] Tan SM, Zhang Y, Connelly KA, Gilbert RE, Kelly DJ (2010). Targeted inhibition of activin receptor-like kinase 5 signaling attenuates cardiac dysfunction following myocardial infarction. Am J Physiol Heart Circ Physiol.

[26] Sun Y, Zhang JQ, Zhang J, Ramires FJ (1998). Angiotensin II, transforming growth factor-beta1 and repair in the infarcted heart. J Mol Cell Cardiol.

[27] Yu CM, Tipoe GL, Wing-Hon Lai K, Lau CP (2001). Effects of combination of angiotensin-converting enzyme inhibitor and angiotensin receptor antagonist on inflammatory cellular infiltration and myocardial interstitial fibrosis after acute myocardial infarction. J Am Coll Cardiol.

[28] Campbell SE, Katwa LC (1997). Angiotensin II stimulated expression of transforming growth factor-beta1 in cardiac fibroblasts and myofibroblasts. J Mol Cell Cardiol.

[29] Gao X, He X, Luo B, Peng L, Lin J, Zuo Z (2009). Angiotensin II increases collagen I expression via transforming growth factor-beta1 and extracellular signal-regulated kinase in cardiac fibroblasts. Eur J Pharmacol.

[30] Ma F, Li Y, Jia L, Han Y, Cheng J, Li H (2012). Macrophage-stimulated cardiac fibroblast production of IL-6 is essential for TGF beta/Smad activation and cardiac fibrosis induced by angiotensin II. PLoS One.

[31] De Mello WC, Specht P (2006). Chronic blockade of angiotensin II AT1-receptors increased cell-to-cell communication, reduced fibrosis and improved impulse propagation in the failing heart. J Renin Angiotensin Aldosterone Syst.

[32] Shibasaki Y, Nishiue T, Masaki H, Tamura K, Matsumoto N, Mori Y (2005). Impact of the angiotensin II receptor antagonist, losartan, on myocardial fibrosis in patients with end-stage renal disease: assessment by ultrasonic integrated backscatter and biochemical markers. Hypertens Res.

[33] Ortiz LA, Lasky J, Gozal E, Ruiz V, Lungarella G, Cavarra E (2001). Tumor necrosis factor receptor deficiency alters matrix metalloproteinase 13/tissue inhibitor of metalloproteinase 1 expression in murine silicosis. Am J Respir Crit Care Med.

[34] Westermann D, Rutschow S, Van Linthout S, Linderer A, Bucker-Gartner C, Sobirey M (2006). Inhibition of p38 mitogen-activated protein kinase attenuates left ventricular dysfunction by mediating pro-inflammatory cardiac cytokine levels in a mouse model of diabetes mellitus. Diabetologia.

[35] Wilson SS, Ayaz SI, Levy PD (2015). Relaxin: a novel agent for the treatment of acute heart failure. Pharmacotherapy.

[36] Turner NA, Porter KE, Smith WH, White HL, Ball SG, Balmforth AJ (2003). Chronic beta2-adrenergic receptor stimulation increases proliferation of human cardiac fibroblasts via an autocrine mechanism. Cardiovasc Res.

[37] Porter KE, Turner NA, O’Regan DJ, Balmforth AJ, Ball SG (2004). Simvastatin reduces human atrial myofibroblast proliferation independently of cholesterol lowering via inhibition of RhoA. Cardiovasc Res.

[38] Blackburn NJ, Sofrenovic T, Kuraitis D, Ahmadi A, McNeill B, Deng C (2015). Timing underpins the benefits associated with injectable collagen biomaterial therapy for the treatment of myocardial infarction. Biomaterials.

[39] Deng B, Shen L, Wu Y, Shen Y, Ding X, Lu S (2015). Delivery of alginate-chitosan hydrogel promotes endogenous repair and preserves cardiac function in rats with myocardial infarction. J Biomed Mater Res A.

[40] Bridges AW, García AJ (2008). Anti-inflammatory polymeric coatings for implantable biomaterials and devices. J Diabetes Sci Technol.

[41] Wang F, Guan J (2010). Cellular cardiomyoplasty and cardiac tissue engineering for myocardial therapy. Adv Drug Deliv Rev.

[42] Wang RM, Christman KL (2016). Decellularized myocardial matrix hydrogels: In basic research and preclinical studies. Adv Drug Deliv Rev.

[43] Turner NA (2015). Inflammatory and fibrotic responses of cardiac fibroblasts to myocardial damage associated molecular patterns (DAMPs). J Mol Cell Cardiol.

[44] Nakamura T, Matsumoto K, Mizuno S, Sawa Y, Matsuda H, Nakamura T (2005). Hepatocyte growth factor prevents tissue fibrosis, remodeling, and dysfunction in cardiomyopathic hamster hearts. Am J Physiol Heart Circ Physiol.

[45] McDade JK, Brennan-Pierce EP, Ariganello MB, Labow RS, Michael LJ (2013). Interactions of U937 macrophage-like cells with decellularized pericardial matrix materials: influence of crosslinking treatment. Acta Biomater.

[46] Sonnenberg SB, Rane AA, Liu CJ, Rao N, Agmon G, Suarez S (2015). Delivery of an engineered HGF fragment in an extracellular matrix-derived hydrogel prevents negative LV remodeling post-myocardial infarction. Biomaterials.

[47] Yoshizumi T, Zhu Y, Jiang H, D’Amore A, Sakaguchi H, Tchao J (2016). Timing effect of intramyocardial hydrogel injection for positively impacting left ventricular remodeling after myocardial infarction. Biomaterials.

[48] Sutton MG, Sharpe N (2000). Left ventricular remodeling after myocardial infarction: pathophysiology and therapy. Circulation.

[49] Lahera V, Cachofeiro V, de Las Heras N (2011). Interplay of hypertension, inflammation, and angiotensin II. Am J Hypertens.

[50] Haudek SB, Cheng J, Du J, Wang Y, Hermosillo-Rodriguez J, Trial J (2010). Monocytic fibroblast precursors mediate fibrosis in angiotensin-II-induced cardiac hypertrophy. J Mol Cell Cardiol.

[51] Bodiga S, Zhong JC, Wang W, Basu R, Lo J, Liu GC (2011). Enhanced susceptibility to biomechanical stress in ACE2 null mice is prevented by loss of the p47(phox) NADPH oxidase subunit. Cardiovasc Res.

[52] Holmes JW, Borg TK, Covell JW (2005). Structure and mechanics of healing myocardial infarcts. Annu Rev Biomed Eng.

[53] Vu TD, Pal SN, Ti LK, Martinez EC, Rufaihah AJ, Ling LH (2015). An autologous platelet-rich plasma hydrogel compound restores left ventricular structure, function and ameliorates adverse remodeling in a minimally invasive large animal myocardial restoration model: a translational approach: Vu and Pal “Myocardial Repair: PRP, Hydrogel and Supplements”. Biomaterials.

[54] Kobayashi H, Minatoguchi S, Yasuda S, Bao N, Kawamura I, Iwasa M (2008). Post-infarct treatment with an erythropoietin-gelatin hydrogel drug delivery system for cardiac repair. Cardiovasc Res.

[55] Taniyama Y, Morishita R, Aoki M, Hiraoka K, Yamasaki K, Hashiya N (2002). Angiogenesis and antifibrotic action by hepatocyte growth factor in cardiomyopathy. Hypertension.

[56] Futamatsu H, Suzuki J, Mizuno S, Koga N, Adachi S, Kosuge H (2005). Hepatocyte growth factor ameliorates the progression of experimental autoimmune myocarditis: a potential role for induction of T helper 2 cytokines. Circ Res.

[57] Nakamura T, Sakai K, Nakamura T, Matsumoto K (2011). Hepatocyte growth factor twenty years on: Much more than a growth factor. J Gastroenterol Hepatol.

[58] Ueda H, Nakamura T, Matsumoto K, Sawa Y, Matsuda H, Nakamura T (2001). A potential cardioprotective role of hepatocyte growth factor in myocardial infarction in rats. Cardiovasc Res.

[59] Nakamura T, Mizuno S, Matsumoto K, Sawa Y, Matsuda H, Nakamura T (2000). Myocardial protection from ischemia/reperfusion injury by endogenous and exogenous HGF. J Clin Invest.

[60] Li Y, Takemura G, Kosai K, Yuge K, Nagano S, Esaki M (2003). Postinfarction treatment with an adenoviral vector expressing hepatocyte growth factor relieves chronic left ventricular remodeling and dysfunction in mice. Circulation.

[61] Nakano J, Marui A, Muranaka H, Masumoto H, Noma H, Tabata Y (2014). Effects of hepatocyte growth factor in myocarditis rats induced by immunization with porcine cardiac myosin. Interact Cardiovasc Thorac Surg.

[62] Ross J, Gherardi E, Mallorqui-Fernandez N, Bocci M, Sobkowicz A, Rees M (2012). Protein engineered variants of hepatocyte growth factor/scatter factor promote proliferation of primary human hepatocytes and in rodent liver. Gastroenterology.

[63] Jones DS, Tsai PC, Cochran JR (2011). Engineering hepatocyte growth factor fragments with high stability and activity as Met receptor agonists and antagonists. Proc Natl Acad Sci U S A.

[64] Liu CJ, Jones DS, Tsai PC, Venkataramana A, Cochran JR (2014). An engineered dimeric fragment of hepatocyte growth factor is a potent c-MET agonist. FEBS Lett.

[65] Ruvinov E, Harel-Adar T, Cohen S (2011). Bioengineering the infarcted heart by applying bio-inspired materials. J Cardiovasc Transl Res.

[66] Ruvinov E, Leor J, Cohen S (2011). The promotion of myocardial repair by the sequential delivery of IGF-1 and HGF from an injectable alginate biomaterial in a model of acute myocardial infarction. Biomaterials.

[67] Salimath AS, Phelps EA, Boopathy AV, Che PL, Brown M, Garcia AJ (2012). Dual delivery of hepatocyte and vascular endothelial growth factors via a protease-degradable hydrogel improves cardiac function in rats. PLoS One.

[68] Samuel SM, Akita Y, Paul D, Thirunavukkarasu M, Zhan L, Sudhakaran PR (2010). Coadministration of adenoviral vascular endothelial growth factor and angiopoietin-1 enhances vascularization and reduces ventricular remodeling in the infarcted myocardium of type 1 diabetic rats. Diabetes.

[69] Zentilin L, Puligadda U, Lionetti V, Zacchigna S, Collesi C, Pattarini L (2010). Cardiomyocyte VEGFR-1 activation by VEGF-B induces compensatory hypertrophy and preserves cardiac function after myocardial infarction. FASEB J.

[70] Ishiguro S, Akasaka Y, Kiguchi H, Suzuki T, Imaizumi R, Ishikawa Y, et al. Basic fibroblast growth factor induces down-regulation of alpha-smooth muscle actin and reduction of myofibroblast areas in open skin wounds. Wound Repair Regen. 2009;17:617–25. 10.1111/j.1524-475X.2009.00511.x 19614927

[71] Shirakihara T, Horiguchi K, Miyazawa K, Ehata S, Shibata T, Morita I (2011). TGF-beta regulates isoform switching of FGF receptors and epithelial-mesenchymal transition. EMBO J.

[72] Cushing MC, Mariner PD, Liao JT, Sims EA, Anseth KS (2008). Fibroblast growth factor represses Smad-mediated myofibroblast activation in aortic valvular interstitial cells. Faseb J.

[73] Svystonyuk DA, Ngu JM, Mewhort HE, Lipon BD, Teng G, Guzzardi DG (2015). Fibroblast growth factor-2 regulates human cardiac myofibroblast-mediated extracellular matrix remodeling. J Transl Med.

[74] Santiago JJ, McNaughton LJ, Koleini N, Ma X, Bestvater B, Nickel BE (2014). High molecular weight fibroblast growth factor-2 in the human heart is a potential target for prevention of cardiac remodeling. PLoS One.

[75] Souders CA, Bowers SL, Baudino TA (2009). Cardiac fibroblast: the renaissance cell. Circ Res.

[76] Banquet S, Gomez E, Nicol L, Edwards-Levy F, Henry JP, Cao R (2011). Arteriogenic therapy by intramyocardial sustained delivery of a novel growth factor combination prevents chronic heart failure. Circulation.

[77] Fan YH, Dong H, Pan Q, Cao YJ, Li H, Wang HC (2011). Notch signaling may negatively regulate neonatal rat cardiac fibroblast-myofibroblast transformation. Physiol Res.

[78] Boopathy AV, Martinez MD, Smith AW, Brown ME, Garcia AJ, Davis ME (2015). Intramyocardial delivery of Notch ligand-containing hydrogels improves cardiac function and angiogenesis following infarction. Tissue Eng Part A.

[79] Forrester JS, Makkar RR, Marban E (2009). Long-term outcome of stem cell therapy for acute myocardial infarction: right results, wrong reasons. J Am Coll Cardiol.

[80] Don CW, Murry CE (2013). Improving survival and efficacy of pluripotent stem cell-derived cardiac grafts. J Cell Mol Med.

[81] Tang YL, Wang YJ, Chen LJ, Pan YH, Zhang L, Weintraub NL (2013). Cardiac-derived stem cell-based therapy for heart failure: progress and clinical applications. Exp Biol Med (Maywood).

[82] Garbern JC, Lee RT (2013). Cardiac stem cell therapy and the promise of heart regeneration. Cell Stem Cell.

[83] Rosen MR, Myerburg RJ, Francis DP, Cole GD, Marban E (2014). Translating stem cell research to cardiac disease therapies: pitfalls and prospects for improvement. J Am Coll Cardiol.

[84] van Berlo JH, Molkentin JD (2014). An emerging consensus on cardiac regeneration. Nat Med.

[85] Nagaya N, Kangawa K, Itoh T, Iwase T, Murakami S, Miyahara Y (2005). Transplantation of mesenchymal stem cells improves cardiac function in a rat model of dilated cardiomyopathy. Circulation.

[86] Gnecchi M, He H, Noiseux N, Liang OD, Zhang L, Morello F (2006). Evidence supporting paracrine hypothesis for Akt-modified mesenchymal stem cell-mediated cardiac protection and functional improvement. FASEB J.

[87] Ripa RS, Haack-Sorensen M, Wang Y, Jorgensen E, Mortensen S, Bindslev L (2007). Bone marrow derived mesenchymal cell mobilization by granulocyte-colony stimulating factor after acute myocardial infarction: results from the Stem Cells in Myocardial Infarction (STEMMI) trial. Circulation.

[88] Hare JM, Traverse JH, Henry TD, Dib N, Strumpf RK, Schulman SP (2009). A randomized, double-blind, placebo-controlled, dose-escalation study of intravenous adult human mesenchymal stem cells (prochymal) after acute myocardial infarction. J Am Coll Cardiol.

[89] Traverse JH, McKenna DH, Harvey K, Jorgenso BC, Olson RE, Bostrom N (2010). Results of a phase 1, randomized, double-blind, placebo-controlled trial of bone marrow mononuclear stem cell administration in patients following ST-elevation myocardial infarction. Am Heart J.

[90] Duran JM, Makarewich CA, Sharp TE, Starosta T, Zhu F, Hoffman NE (2013). Bone-derived stem cells repair the heart after myocardial infarction through transdifferentiation and paracrine signaling mechanisms. Circ Res.

[91] Toma C, Pittenger MF, Cahill KS, Byrne BJ, Kessler PD (2002). Human mesenchymal stem cells differentiate to a cardiomyocyte phenotype in the adult murine heart. Circulation.

[92] Balana B, Nicoletti C, Zahanich I, Graf EM, Christ T, Boxberger S (2006). 5-Azacytidine induces changes in electrophysiological properties of human mesenchymal stem cells. Cell Res.

[93] Qian Q, Qian H, Zhang X, Zhu W, Yan YM, Ye SQ (2012). 5-azacytidine induces cardiac differentiation of human umbilical cord-derived mesenchymal stem cells by activating extracellular regulated kinase. Stem Cells Dev.

[94] Wang CC, Chen CH, Lin WW, Hwang SM, Hsieh PCH, Lai PH (2008). Direct intramyocardial injection of mesenchymal stem cell sheet fragments improves cardiac functions after infarction. Cardiovasc Res.

[95] Martinez EC, Kofidis T (2011). Adult stem cells for cardiac tissue engineering. J Mol Cell Cardiol.

[96] Perin EC, Tian M, Marini FC, Silva GV, Zheng Y, Baimbridge F (2011). Imaging long-term fate of intramyocardially implanted mesenchymal stem cells in a porcine myocardial infarction model. Plos One.

[97] Pittenger MF, Martin BJ (2004). Mesenchymal stem cells and their potential as cardiac therapeutics. Circ Res.

[98] Mias C, Lairez O, Trouche E, Roncalli J, Calise D, Seguelas MH (2009). Mesenchymal stem cells promote matrix metalloproteinase secretion by cardiac fibroblasts and reduce cardiac ventricular fibrosis after myocardial infarction. Stem Cells.

[99] Zuo S, Jones WK, Li HX, He ZS, Pasha ZS, Yang YT (2012). Paracrine effect of Wnt11-overexpressing mesenchymal stem cells on ischemic injury. Stem Cells Dev.

[100] Mazo M, Planat-Benard V, Abizanda G, Pelacho B, Leobon B, Gavira JJ (2008). Transplantation of adipose derived stromal cells is associated with functional improvement in a rat model of chronic myocardial infarction. Eur J Heart Fail.

[101] Mazo M, Hernandez S, Gavira JJ, Abizanda G, Arana M, Lopez-Martinez T (2012). Treatment of reperfused ischemia with adipose-derived stem cells in a preclinical Swine model of myocardial infarction. Cell Transplant.

[102] Shevchenko EK, Makarevich PI, Tsokolaeva ZI, Boldyreva MA, Sysoeva VY, Tkachuk VA (2013). Transplantation of modified human adipose derived stromal cells expressing VEGF165 results in more efficient angiogenic response in ischemic skeletal muscle. J Transl Med.

[103] Rigol M, Solanes N, Roura S, Roque M, Novensa L, Dantas AP (2014). Allogeneic adipose stem cell therapy in acute myocardial infarction. Eur J Clin Invest.

[104] Hosoda T, Zheng H, Cabral-da-Silva M, Sanada F, Ide-Iwata N, Ogorek B (2011). Human cardiac stem cell differentiation is regulated by a mircrine mechanism. Circulation.

[105] Chugh AR, Beache GM, Loughran JH, Mewton N, Elmore JB, Kajstura J (2012). Administration of cardiac stem cells in patients with ischemic cardiomyopathy: the SCIPIO trial: surgical aspects and interim analysis of myocardial function and viability by magnetic resonance. Circulation.

[106] Bolli R, Tang XL, Sanganalmath SK, Rimoldi O, Mosna F, Abdel-Latif A (2013). Intracoronary delivery of autologous cardiac stem cells improves cardiac function in a porcine model of chronic ischemic cardiomyopathy. Circulation.

[107] Latham N, Ye B, Jackson R, Lam BK, Kuraitis D, Ruel M (2013). Human blood and cardiac stem cells synergize to enhance cardiac repair when cotransplanted into ischemic myocardium. Circulation.

[108] Williams AR, Hatzistergos KE, Addicott B, McCall F, Carvalho D, Suncion V (2013). Enhanced effect of combining human cardiac stem cells and bone marrow mesenchymal stem cells to reduce infarct size and to restore cardiac function after myocardial infarction. Circulation.

[109] Spater D, Abramczuk MK, Buac K, Zangi L, Stachel MW, Clarke J (2013). A HCN4+ cardiomyogenic progenitor derived from the first heart field and human pluripotent stem cells. Nat Cell Biol.

[110] Nsair A, Schenke-Layland K, Van Handel B, Evseenko D, Kahn M, Zhao P (2012). Characterization and therapeutic potential of induced pluripotent stem cell-derived cardiovascular progenitor cells. PLoS One.

[111] Hudson J, Titmarsh D, Hidalgo A, Wolvetang E, Cooper-White J (2012). Primitive cardiac cells from human embryonic stem cells. Stem Cells Dev.

[112] Smith RR, Barile L, Cho HC, Leppo MK, Hare JM, Messina E (2007). Regenerative potential of cardiosphere-derived cells expanded from percutaneous endomyocardial biopsy specimens. Circulation.

[113] Davis DR, Zhang Y, Smith RR, Cheng K, Terrovitis J, Malliaras K (2009). Validation of the cardiosphere method to culture cardiac progenitor cells from myocardial tissue. PLoS One.

[114] Johnston PV, Sasano T, Mills K, Evers R, Lee ST, Smith RR (2009). Engraftment, differentiation, and functional benefits of autologous cardiosphere-derived cells in porcine ischemic cardiomyopathy. Circulation.

[115] Chimenti I, Smith RR, Li TS, Gerstenblith G, Messina E, Giacomello A (2010). Relative roles of direct regeneration versus paracrine effects of human cardiosphere-derived cells transplanted into infarcted mice. Circ Res.

[116] Mishra R, Vijayan K, Colletti EJ, Harrington DA, Matthiesen TS, Simpson D (2011). Characterization and functionality of cardiac progenitor cells in congenital heart patients. Circulation.

[117] Li TS, Cheng K, Malliaras K, Smith RR, Zhang Y, Sun B (2012). Direct comparison of different stem cell types and subpopulations reveals superior paracrine potency and myocardial repair efficacy with cardiosphere-derived cells. J Am Coll Cardiol.

[118] Maxeiner H, Mufti S, Krehbiehl N, Dulfer F, Helmig S, Schneider J (2014). Interleukin-6 contributes to the paracrine effects of cardiospheres cultured from human, murine and rat hearts. J Cell Physiol.

[119] Xie Y, Ibrahim A, Cheng K, Wu Z, Liang W, Malliaras K (2014). Importance of cell-cell contact in the therapeutic benefits of cardiosphere-derived cells. Stem Cells.

[120] Davis DR, Kizana E, Terrovitis J, Barth AS, Zhang YQ, Smith RR (2010). Isolation and expansion of functionally-competent cardiac progenitor cells directly from heart biopsies. J Mol Cell Cardiol.

[121] Davis DR, Zhang YQ, Smith RR, Cheng K, Terrovitis J, Malliaras K, et al. Validation of the cardiosphere method to culture cardiac progenitor cells from myocardial tissue. Plos One. 2009;4. 10.1371/journal.pone.0007195 PMC2745677 19779618

[122] Lee ST, White AJ, Matsushita S, Malliaras K, Steenbergen C, Zhang Y (2011). Intramyocardial injection of autologous cardiospheres or cardiosphere-derived cells preserves function and minimizes adverse ventricular remodeling in pigs with heart failure post-myocardial infarction. J Am Coll Cardiol.

[123] Sun CK, Zhen YY, Leu S, Tsai TH, Chang LT, Sheu JJ (2014). Direct implantation versus platelet-rich fibrin-embedded adipose-derived mesenchymal stem cells in treating rat acute myocardial infarction. Int J Cardiol.

[124] Arana M, Gavira JJ, Pena E, Gonzalez A, Abizanda G, Cilla M (2014). Epicardial delivery of collagen patches with adipose-derived stem cells in rat and minipig models of chronic myocardial infarction. Biomaterials.

[125] Ohnishi S, Sumiyoshi H, Kitamura S, Nagaya N (2007). Mesenchymal stem cells attenuate cardiac fibroblast proliferation and collagen synthesis through paracrine actions. FEBS Lett.

[126] Xia Y, Zhu K, Lai H, Lang M, Xiao Y, Lian S (2015). Enhanced infarct myocardium repair mediated by thermosensitive copolymer hydrogel-based stem cell transplantation. Exp Biol Med (Maywood).

[127] Ceccaldi C, Bushkalova R, Alfarano C, Lairez O, Calise D, Bourin P (2014). Evaluation of polyelectrolyte complex-based scaffolds for mesenchymal stem cell therapy in cardiac ischemia treatment. Acta Biomater.

[128] Fiumana E, Pasquinelli G, Foroni L, Carboni M, Bonafe F, Orrico C (2013). Localization of mesenchymal stem cells grafted with a hyaluronan-based scaffold in the infarcted heart. J Surg Res.

[129] Engler AJ, Carag-Krieger C, Johnson CP, Raab M, Tang HY, Speicher DW (2008). Embryonic cardiomyocytes beat best on a matrix with heart-like elasticity: scar-like rigidity inhibits beating. J Cell Sci.

[130] Nelson DM, Baraniak PR, Ma Z, Guan J, Mason NS, Wagner WR (2011). Controlled release of IGF-1 and HGF from a biodegradable polyurethane scaffold. Pharm Res.

[131] Tamama K, Kawasaki H, Kerpedjieva SS, Guan J, Ganju RK, Sen CK (2011). Differential roles of hypoxia inducible factor subunits in multipotential stromal cells under hypoxic condition. J Cell Biochem.

[132] Guo X, Elliott CG, Li Z, Xu Y, Hamilton DW, Guan J (2012). Creating 3D angiogenic growth factor gradients in fibrous constructs to guide fast angiogenesis. Biomacromolecules.

[133] Li Z, Guo X, Guan J (2012). A thermosensitive hydrogel capable of releasing bFGF for enhanced differentiation of mesenchymal stem cell into cardiomyocyte-like cells under ischemic conditions. Biomacromolecules.

[134] Wang F, Li Z, Khan M, Tamama K, Kuppusamy P, Wagner WR (2010). Injectable, rapid gelling and highly flexible hydrogel composites as growth factor and cell carriers. Acta Biomater.

[135] Wang F, Li Z, Tamama K, Sen CK, Guan J (2009). Fabrication and characterization of prosurvival growth factor releasing, anisotropic scaffolds for enhanced mesenchymal stem cell survival/growth and orientation. Biomacromolecules.

[136] Li Z, Guo X, Guan J (2012). An oxygen release system to augment cardiac progenitor cell survival and differentiation under hypoxic condition. Biomaterials.

